# Gluten and Functional Abdominal Pain Disorders in Children

**DOI:** 10.3390/nu10101491

**Published:** 2018-10-12

**Authors:** Alejandro Llanos-Chea, Alessio Fasano

**Affiliations:** 1 Mucosal Immunology and Biology Research Center, Division of Pediatric Gastroenterology and Nutrition, Massachusetts General Hospital, 114 16th Street (M/S 114-3503), Charlestown, Boston, MA 33131, USA; allanos-chea@mgh.harvard.edu; 2 Department of Pediatrics, Harvard Medical School, Boston, MA 33131, USA; 3 Division of Pediatric Gastroenterology, Hepatology & Nutrition, University of Miami Miller School of Medicine, Miami, FL 33136, USA

**Keywords:** pediatric functional abdominal pain disorders, gluten, wheat, celiac disease, non-celiac gluten sensitivity

## Abstract

In children, functional gastrointestinal disorders (FGIDs) are common at all ages. Consumption of certain foods, particularly gluten, is frequently associated with the development and persistence of FGIDs and functional abdominal pain disorders (FAPDs) in adults and children. However, this association is not well defined. Even without a diagnosis of celiac disease (CD), some people avoid gluten or wheat in their diet since it has been shown to trigger mostly gastrointestinal symptoms in certain individuals, especially in children. The incidence of conditions such as non-celiac gluten sensitivity (NCGS) is increasing, particularly in children. On the other hand, CD is a chronic, autoimmune small intestinal enteropathy with symptoms that can sometimes be mimicked by FAPD. It is still unclear if pediatric patients with irritable bowel syndrome (IBS) are more likely to have CD. Abdominal, pain-associated FGID in children with CD does not seem to improve on a gluten-free diet. The threshold for gluten tolerance in patients with NCGS is unknown and varies among subjects. Thus, it is challenging to clearly distinguish between gluten exclusion and improvement of symptoms related solely to functional disorders.

## 1. Introduction

The association between gluten and abdominal pain is broad and can be attributed to many factors [1,2,3,4]. Nevertheless, the association between gluten and functional abdominal pain disorders (FAPDs) is not well defined. In children, functional gastrointestinal disorders (FGIDs) are common at all ages [5,6,7,8,9]. FAPD is a relatively new term that was recently defined in the Rome IV criteria in 2016 (Table 1).

The Rome criteria are defined by experts who use evidence, when available, and clinical experience or consensus when lacking scientific data for certain conditions. The pediatric section of this criteria recommends diagnosing FGID in children and adolescents using symptom-based guidelines. The Rome IV criteria does mention that FGIDs are not necessarily diagnoses of exclusion, and that other medical conditions can coexist with FGIDs [11,12]. The term, “abdominal pain-related functional gastrointestinal disorders” was changed to “functional abdominal pain disorders” (FAPDs) in 2016 and a new term, “functional abdominal pain—not otherwise specified (FAP-NOS)” for pediatric patients, was added for patients who do not fit the criteria for irritable bowel syndrome (IBS), functional dyspepsia, or abdominal migraine [10]. 

Many studies have described the frequency of FGIDs in children [13,14,15,16,17,18]. However, these studies have used the definitions described in the Rome III criteria. For example, dyspeptic symptoms have been reported in 5–10% of healthy adolescents in the northeastern United States [15]. As per parent reports, IBS prevalence in children in the United States can range from 1.2% to 2.9% [14,17] and, as per school-based studies, the prevalence in Colombia and Sri Lanka has been reported to be 4.9% and 5.4%, respectively [13,16]. Depending on the criteria used for diagnosis of abdominal migraine, its frequency can vary between 1% and 23% [13,14,17,18]. The Rome III criteria described functional abdominal pain (FAP) and FAP syndrome (FAPS) [10]. As per prior reports, 38% of school-aged children report weekly abdominal pain. A Colombian cross-sectional study found a prevalence that ranged from 1.7% to 2% for FAP and 0.3% to 1.4% for FAPS in children [13].

Robin et al. reported a U.S. prevalence of FGIDs in children 0–18 years old, according to the newly established Rome IV diagnostic criteria. Data was collected using the Rome IV Pediatric Diagnostic Questionnaire (RIV-PDQ), which has been validated in adults but not in patients younger than 18 years. From 959 children ages 4 years or older included in the study, symptom-based FGID criteria were met by 25% of children and adolescents, with functional dyspepsia (postprandial distress syndrome) being the most common FAPD, being present in 7.2% of children [19]. Similarly, Saps et al. completed a cross-sectional study using a Spanish version of the RIV-PDQ that was applied to school-aged children (8 to 18 years) in Colombia. After exclusions, 3567 children completed the study; 21.2% met the Rome IV criteria for FGIDs, and 8.2% had one FAPD. In Colombia, the prevalence of FGIDs was significantly lower (*p* = 0.004) than in a previous study using Rome III questionnaires, at 23.7% [20]. As with the U.S.-based study, functional dyspepsia (postprandial distress syndrome) was the most common FAPD in 2.7% of children; it was followed closely by FAP-NOS (2.4%) and IBS (2.3%) [19,20]. It is of note that rates of functional dyspepsia (postprandial distress syndrome) and IBS were much higher in U.S. pediatric participants than Colombian children (Table 2). Due to the lack of multicenter, large, international comparative studies, the reason for the difference in prevalence between various geographic regions is currently unknown [21]. In addition, these differences can be influenced by population variability in regard to social and psychosocial factors (i.e., divorce, mental illness and other social stressors). These factors are involved in the pathogenesis of FGIDs as described by the biopsychosocial model [22,23]. Finally, the U.S.-based study was conducted using a maternal online survey, and the study from Colombia collected data from children at their schools. Regardless of method, neither study author could completely assure recall accuracy [24].

## 2. Pathogenesis of FGID and FAPD

Functional gastrointestinal disorders are mostly considered the sum of many complex factors that interact between them, including early life, genetics, and biological, psychological, environmental, and social factors [22,23]. 

**Figure 1:**
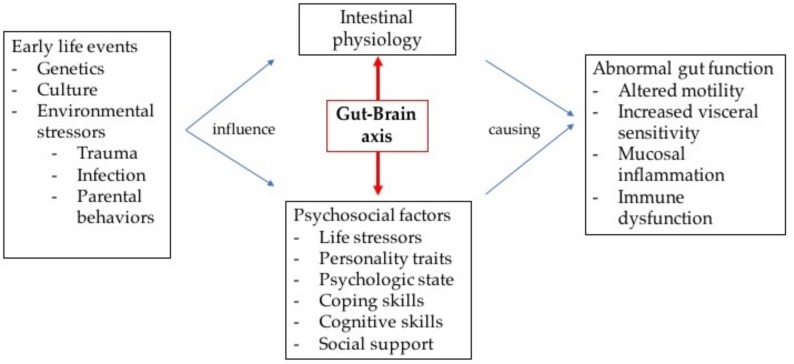
Biopsychosocial approach and gut–brain axis [22,25,27,28,29].

Using the biopsychosocial approach allows the clinician to better understand FGIDs and establish a clinical framework to address the variability and complexity of these patients [22,23]. Early life events influence psychosocial factors and intestinal physiology, which will interact as part of the gut–brain axis. Using neurotransmitters, this axis bidirectionally transmits signals from the brain’s cognitive and emotional centers to the gastrointestinal (GI) tract [25]. Several motor, sensory, autonomic, immune and endocrine functions are affected through direct connections between the visceral muscles, other end-organ structures, and the central nervous system (CNS) [26]. Psychosocial factors will modulate the patient’s pain experience and symptom behavior (Figure 1) [22]. 

The gut microbiome has been recognized as an important player in the pathogenesis of FGID. The microbiome contains approximately 100 times as many genes as the human genome, and it is composed of approximately 10^13^ to 10^14^ microorganisms with 500 to 1000 different species [30]. Under physiological circumstances, intestinal bacteria maintain a homeostatic relationship with host mucosa without inducing the systemic immune system, while in pathological conditions this balance seems to be lost due to dysbiosis [31]. For example, fecal and intestinal microbial diversity differences are found among healthy and IBS patients [32]. These alterations lead to enhanced intestinal permeability, mucosal immune activation, altered gut motility, and visceral hypersensitivity [32,33,34,35,36,37,38]. Nevertheless, the data are conflicting when comparing the composition and difference between the intestinal microbiome and chronic abdominal pain in adults and children with IBS [39].

The emerging concept of the microbiome–gut–brain axis [39,40] is described as the control of the GI and neurological function by the connection of the microbiota, gut, and brain [40]. The CNS modulates the GI tract via the autonomic nervous system (ANS). The microbiota is influenced by the hypothalamus–pituitary–adrenal (HPA) axis through changes in the environment, i.e., secretion of acid, mucus, regional motility, gut permeability, and host-enteric microbiota signaling through short chain fatty acids and neuroactive substances [41,42,43]. 

More recently food and diet, and their effect on intestinal microbiota, have been associated with FGIDs. Milk and carbohydrates, respectively, are the most common foods avoided by patients with IBS [44,45]. Certain food restrictions could be beneficial as a result of reduced osmotic effects or alterations in gut mucosa [46,47]; however, results are inconsistent [48]. Wheat is produced in more than 25,000 different cultivars. Its simplicity for cultivation in different climates, and its nutritional value, palatability, and other factors make it the most widely grown crop worldwide. Wheat is processed in many food and drinks; and gluten is used as an additive in foods and cosmetics [4]. Even though the reason is not clear and there is concern in terms of long-term health effects, over the last decade the gluten-free market has grown rapidly. By 2017 the gluten-free market in the United States was estimated at US$6 billion [49,50]. The increased number of people who avoid gluten is due to increasing concerns about gluten intolerance or celiac disease (CD) [51]. We will discuss the direct effect of gluten in FAPDs not only as a food but also as part of recognized conditions such as CD and non-celiac gluten sensitivity (NCGS).

## 3. Gluten as a Factor in FAPDs

The epidemiology of gluten-related FAPDs has not been yet reported. Among the conditions included in FAPDs, IBS has been the one most studied with regards to its relationship to diet and gluten consumption [1]. More than 60% of adult patients with IBS develop bloating and abdominal pain within 15 minutes to a few hours after consumption of certain foods [44]. In a double-blind, randomized placebo-controlled trial, patients with IBS and self-reported gluten intolerance with a negative diagnosis for CD received either gluten or a placebo. Thirty-two percent of patients in the gluten-exposed group reported adequate symptom control versus 60% of the placebo group, suggesting that patients with IBS could react to gluten despite the lack of gluten intolerance diagnosis [52]. Similar studies in children are lacking [53].

There is an efficient and coordinated system that responds to food ingestion with digestion, absorption of nutrients and waste expulsion. Certain foods can affect some patients due to various mechanisms that include intolerance, allergy, and/or hypersensitivity [54]. Wheat in the diet plays an important role by worsening symptoms in patients with IBS [55]. Gluten, found in wheat, rye, and barley, is a group of immunogenic proteins that is known to cause CD, an autoimmune disease, in people with genetic predisposition [56]. 

Gut proteases do not completely degrade gluten proteins, leading to the production of several non-digested peptides. It has been proposed that, even in the absence of CD, these peptides can cause mild gut immune and/or functional abnormalities in a subgroup of patients with IBS [57]. Mouse models with sensitization to gluten, in the absence of CD, have described altered smooth muscle contractility and an abnormal immune reaction associated with IBS [1]. In addition, α-amylase/trypsin inhibitors (ATIs) and wheat lectin agglutinin, other protein components of wheat, have been demonstrated to stimulate pathways of innate immunity [2,58]. The role of ATIs in IBS is not yet known, and its mechanisms are different from those proposed for gluten with the option to co-exist or act in a synergistic matter [1]. Wheat also contains fructans, which are carbohydrates that are poorly absorbed in the small intestine, as well as other fermentable oligosaccharides, disaccharides, mono-saccharides, and polyols (FODMAPs) [55]. The implication of FODMAPs in FAPD is not discussed in this review.

Even in the absence of CD, gluten has been hypothesized to trigger GI symptoms. Mice sensitized to gluten have an increased release of acetylcholine from the myenteric plexus. As the main excitatory neurotransmitter in the intestine, acetylcholine increases smooth muscle contractility, ion transport, and water vectorial movements. Sensitization with non-gluten protein did not elicit a similar reaction, and gluten did not cause any mucosal atrophy. This dysfunction was more pronounced in the T-cell response against the human leukocyte antigen (HLA) in DQ8-positive transgenic mice [59]. Monocytes of subjects without CD, as established by negative tissue microscopy and serology, with a positive HLA-DQ2 genetic haplotype, have been shown to release 2–3 fold more interleukin (IL) 8 compared to monocytes from HLA-DQ2 negative individuals [60]. 

**What we know**: IBS symptoms can be present when individuals are exposed to gluten even without a diagnosis of intolerance to gluten. Functional abdominal pain and IBS can be triggered not only by gluten, but also by other components of wheat including ATIs, wheat, lectin, agglutinin, and fructans.

**What we do not know**: The epidemiology of gluten-related FAPD in children, and the genetic predisposition, specifically relying on HLA haplotypes, is unclear.

## 4. Non-Celiac Gluten Sensitivity and FAPD

Even without a diagnosis of CD, approximately 1.7% of Americans avoid gluten or wheat in their diet [61]. Worldwide, 4 to 13% of the general population self-report symptoms related to gluten or wheat ingestion [62,63,64,65,66,67,68,69].

Non-celiac gluten sensitivity (NCGS) has been defined as a condition in which intestinal and/or extra-intestinal symptoms are triggered by gluten ingestion which resolve once gluten is eliminated from the diet, provided that CD and wheat allergy have been ruled out. The innate immune system has been proposed as contributing to the pathogenesis of NCGS, as these patients have been found to have increased intestinal mucosa toll-like receptor (TLR) 2 expression when compared to celiac patients [3]. Pro-inflammatory cytokines and co-stimulatory molecules are expressed following exposure to gliadin, the major component of wheat gluten, in monocytes and dendritic cells [2,59,70,71]. Peptides derived from gliadin could increase IL-15 production and promote enterocyte apoptosis [71].

Whether gluten is really causing symptoms in this NCGS patient population remains the object of an ongoing debate [4]. NCGS is usually categorized as a food sensitivity more than a food intolerance. Food intolerance is defined as abnormal food digestion secondary to the inability to digest nutrients or excessive intake of specific nutrients that would then be only partially digested and absorbed, so leading to GI symptoms related to intestinal microbiota sugar fermentation [72]. Food sensitivities are variable, immune-mediated reactions to specific nutrients that lead to intestinal and/or extra-intestinal clinical manifestations [4]. Based on the above-mentioned definition, it has been proposed that NCGS and IBS are different conditions with overlapping features [4]. In some studies, these two clinical entities have been considered as synonymous, creating major confusion in data interpretation and outcome. A FODMAPs diet can resolve the symptoms of a subgroup of IBS patients, while this diet would not have any effect on true NCGS patients, since FODMAPs do not induce an immune response, and the elimination of gluten-containing food will only minimally decrease the overall intake of FODMAPs [4]. Other proteins unique to gluten-containing cereal have been shown to trigger immune responses, which has resulted in the generation of the alternate term of wheat sensitivity or non-celiac wheat sensitivity (NCWS) [1,4]. 

A subgroup of IBS patients, especially the ones with diarrhea-predominant IBS, could have NCGS with positive HLA-DQ2 and/or HLA-DQ8 genotypes [73]. These patients have breaks in tight junctions, with increased antigen trafficking and activation of immune cells on the lamina propria, but no changes in intestinal transit time or mucosal histology [73,74]. Once gluten is excluded, most patients would experience improved clinical symptoms [74]. Messenger RNA analysis from rectosigmoid biopsies obtained from these patients showed decreased expression of occludin, claudin-1, and zonula occludens-1 [73]. In this IBS population with potential NCGS, there is an increased trafficking of dietary antigens as well as lipopolysaccharide (LPS) from the intestinal lumen to the submucosa due to increased gut permeability. An uncontrolled intestinal influx of LPS induced immunopathology, with the development and progression of low-grade, non-infective, chronic inflammation as detected by increased levels of tumor necrosis factor α and IL-1β [75]. Adaptive immunity cytokines have been found to be normal [76]; however, a study showed elevated small intestine IFN-γ levels when NCGS patients were exposed to gluten [77]. It is debatable if this cytokine was produced by intraepithelial lymphocytes or as part of a T helper 1 adaptive immune response [77]. Furthermore, mucosa permeability is affected by lumen proteases, while prostanoids and histamine released by immune cells seem to enhance activation of neural responses responsible for motor intestinal function and abdominal pain perception [78].

Patients with NCGS have been reported to have different intestinal microbiota when compared to the general population, having a lower proportion of fecal *Lactobacillus* and *Bifidobacterium* and elevated duodenal presence of *Bacteroides* and *Escherichia coli* [79]. Patients with NCGS could present with small intestinal bacterial overgrowth in 9 to 55% of cases, especially in patients non-responsive to a gluten-free diet or with symptom recurrence after an initial response to the gluten-free diet [80].

Due to the lack of validated biomarkers, the prevalence of NCGS/NCWS is not clear in the general population. A study that analyzed the National Health and Nutrition Examination Survey (NHANES) from 2009 to 2010 showed that from 7762 unselected participants aged 6 years old or older, 0.55% of them reported gluten-free diet consumption [81]. It is probable that several of these subjects could have NCGS [4]. Between 2004 and 2010, the Center for Celiac Research at the University of Maryland estimated a 6% prevalence for potential NCGS/NCWS [82], while an Italian multicenter prospective study in 2013 showed 3.19% patients with suspected NCGS/NCWS [83]. Similar to what has been found in IBS, NCGS seems to be more common in females, particularly around the third decade of life, with the diagnosis occurring more often in tertiary centers [55]. Unlike adults, NCGS has been reported to be more common in male than female children [84].

Studies in pediatric patients are almost inexistent. A study in New Zealand reported that 5% of children without a diagnosis of CD avoided gluten-containing foods to prevent GI symptoms and/or non-specific behavioral changes. Only a few children were actually tested for CD with procedures that included small intestine histologic evaluation [68]. There are two prospective series of where NCGS was diagnosed in 12 and 15 cases with clinical symptoms similar to the ones described in adults [84,85].

Clinical symptoms of NCGS/NCWS usually present immediately after the ingestion of gluten-containing foods with resolution after withdrawal and recurrence after challenge [82,86,87]. Gastrointestinal symptoms include bloating, abdominal discomfort, abdominal pain, altered bowel habits, and/or tiredness [88]. IBS is characterized by a similar presentation, but NCGS tends to have more extra-intestinal manifestations that include central and/or peripheral nervous system-associated symptoms, musculoskeletal and skin manifestations, chronic fatigue and “foggy mind” [83]. In children, NCGS mainly manifests with intestinal symptoms like abdominal pain and chronic diarrhea without weight loss [4]. In a small cohort of 15 pediatric patients with NCGS, most common GI symptoms included abdominal pain (80%), chronic diarrhea (73%), bloating (26%), vomiting (20%), and constipation (20%) [84].

NCGS should not only be a diagnosis of exclusion; it is important that CD and wheat allergy are accurately excluded using serologic markers and/or histology [88]. By the time they seek medical care, most patients with NCGS have already established a relationship between symptoms and exposure to gluten [4]. There is no biomarker with sufficient sensitivity and specificity for diagnostic purposes. Diagnostic criteria rely on both the assessment of clinical response to a gluten-free diet and the negative potential influence of gluten consumption after a gluten-free diet period (“nocebo” effect) [1,86]. In the clinical setting, a blind, crossover gluten challenge is difficult and not usually feasible [88].

Duodenal biopsies are normal on a gluten-containing diet, but up to 40% of cases could present with mild elevation of intraepithelial lymphocytes, especially CD3+ immune stain [83,89]. Testing for HLA complex specifically for NCGS/NCWS is not recommended [1,90]. As a potential biomarker, immunoglobulin G (IgG) anti-gliadin antibodies (AGAs) have been proposed for diagnosis of NCGS/NCWS. High antibody titers are detectable in the serum of more than 50% of NCGS/NCWS patients. It is important to mention that AGA are present in other conditions including connective tissue disorders, autoimmune diseases and healthy controls [1]. One study described AGA normalization after following the gluten-free diet for 6 months in almost all patients with NCGS [91].

In pediatrics, there is also an absence of biomarkers. Francavilla et al. described that NCGS children compared to controls tested positive for AGA IgG titers, and positivity for HLA-DQ2 was significantly more common, being present in 66% and 46% of cases, respectively. No differences in nutritional, biochemical or inflammatory markers were found between the children with NCGS and controls. Diagnosis of NCGS in children could be challenging, considering a potential overlap with a delayed allergic reaction with symptoms that develop from one hour to several days after ingestion of wheat proteins. Many of these infants would have negative serum immunoglobulin E (IgE) levels and skin prick tests with absence of circulating wheat protein-specific IgE [84].

**What we know**: NCGS and IBS are different conditions with overlapping features. Adaptative immunity is normal in NCGS cases. Patients with NCGS have altered microbiota and are at increased risk for small intestinal bacterial overgrowth. NCGS is a diagnosis of exclusion.

**What we do not know**: If gluten truly causes symptoms in NGCS patients. There are no validated biomarkers for NCGS. Small case series in pediatrics provide inconclusive evidence for NCGS, but there are reports of children without CD avoiding gluten in their diet.

## 5. Celiac Disease and FAPD

CD is a chronic, autoimmune, small intestinal enteropathy mediated by T lymphocytes immune response, triggered in subjects with genetic predisposition by exposure to dietary gluten [88]. Clinical presentation in older children and adults can include, among other symptoms, abdominal pain, diarrhea, bloating, constipation, and/or weight loss [56]. These symptoms can sometimes be mimicked by symptoms of FAPD. Diarrhea and abdominal pain are more frequently present in individuals with both conditions [10,92]. Due to similarity of symptoms, there can be misdiagnosis in patients who could have CD, as with patients already diagnosed with CD receiving treatment [93]. For that reason, the Rome IV criteria recommend an accurate evaluation for CD in children with symptoms suggestive of diarrhea-predominant IBS, especially in the setting of positive family history for CD [10]. 

It is still unclear if pediatric patients with IBS are more likely to have CD. In adults, CD has been reported to be 3–4 times more common in patients with IBS [94], with 4% of patients initially diagnosed with IBS ultimately being diagnosed with CD [94,95,96,97]. Recent studies report lower frequencies, with 0.4% in Norway [98], 2% in Turkey [99], and 3.2% in Jordan [100]. In addition, adult studies have demonstrated that 35% of patients with CD adherent to a gluten-free diet will continue to have abdominal pain and discomfort, and 22% have persistence of diarrhea [101]. A US-based adult cohort found that 48% of patients diagnosed with CD met the criteria for IBS upon diagnosis, while only 2% continued fulfilling the criteria 6 months after diagnosis. Improvement or resolution of abdominal pain occurred shortly after the implementation of the gluten-free diet in 95% of patients [102]. Contradictory results were reported in a Canadian cohort, indicating symptoms persisted even on an adequate gluten-free diet. After 5 years on a gluten-free diet as treatment for CD, persistent symptoms included diarrhea, abdominal pain and constipation in 22%, 35% and 46% of cases, respectively. Authors proposed a post-inflammatory effect toward FAP as a potential cause [101]. A meta-analysis reported a higher prevalence of biopsy-proven CD and positive serology (IgA anti-gliadin antibodies, endomysial antibodies, and/or tissue transglutaminase antibodies) in adult participants with symptoms suggestive of IBS when compared to healthy controls. Results were not consistent in population-based studies, and among IBS patients in North American studies, there was no increase in odds ratio for any CD test [103]. 

Very limited studies in children have suggested that FAPD and IBS are unlikely to be caused by CD [53]. Nevertheless, a tertiary center, prospective cohort reported that there was a 4-fold increased incidence of CD among children with Rome III criteria diagnosis for IBS [104]. No association has been found between recurrent abdominal pain and the prevalence of anti-endomysial antibody when compared to healthy controls [105]. In the United States, diagnosis of CD was positive for only one of 227 patients with recurrent abdominal pain aged 5 to 18 years [106]. A prospective Italian cohort of children between 4 and 17 years of age, who were followed prospectively for one year after keeping a gluten-free diet, reported that 28% (23 of 82) of participants continued having GI symptoms and fulfilled Rome III criteria for FGIDs. In comparison, only 8.9% (5 of 56) of healthy controls had FGIDs. Anxiety and depression were significantly more present in patients with CD and FGIDs than controls and children with CD without FGIDs (*p* = 0.02). After one year on a gluten-free diet, participants with GI symptoms alone (40.3%) met Rome III criteria for FGIDs significantly more than children with also extra-intestinal symptoms [107]. Psychological factors, but also residual chronic inflammation, were postulated as potential causes, along with the similar explanation by Pulido et al. in adults [101,107]. Functional GI disorders related to post inflammation possibly differ between children and adults. For example, it is still unclear what the mechanisms are by which sometimes acute inflammation secondary to acute gastroenteritis leads to FGIDs, while chronic inflammation for months or years as seen in CD does not trigger FGIDs [108,109].

More recently, Saps et al. conducted a multinational, cross-sectional study in the United States and Italy. Two-hundred-and-eighty-nine children were recruited. The cohort included children with CD on a gluten-free diet for more than 6 months, sibling controls and unrelated controls. For sibling control, the closest sibling of the index case was recruited, ideally of the same sex, with normal tTG-IgA antibody levels and duodenal biopsies if an upper endoscopy had been completed. Abdominal pain-associated FGIDs were present in 8.2% of participants with CD; in 8.2% of sibling controls; and in 2.1% of unrelated individuals. The relative risk for abdominal pain-associated FGIDs was not significantly different among patients with CD on a gluten-free diet and sibling controls (*p* = 1.00). Even though there was a 4-fold increase between these both groups compared to unrelated controls, there still was not statistical difference among them (*p* = 0.06 for both) [110]. Chronic abdominal pain and abdominal pain-associated FGIDs were more common in children from Italy compared to the U.S. participants; 46% and 14%, respectively, in Italy versus 19%, and 2% in the United States. A similar study that compared 46 pediatric patients with CD on a gluten-free diet to sibling controls did not show a difference in abdominal pain and abdominal pain-associated FGIDs between those groups [111]. In adults, a meta-analysis indicated that there is a 5-fold greater risk for patients with CD, irrespective of gluten-free diet adherence, to have IBS-type symptoms when compared to healthy controls [97]. This association has not been studied and it is not clear that lack of consistency among children and adults. It has been proposed that adult patients with CD have chronic duration of symptoms (mean duration 11 to 13 years) [87,101,112,113,114] before diagnosis, which led to a longer time period of intestinal mucosa inflammation [110].

Due to the lack of consistent data among the prevalence of FGIDs in children with CD following a gluten-free diet, there are relevant clinical implications and a treatment dilemma. Options include the avoidance of unnecessary testing or insistence on an improved diet adherence under the premise of a pediatric subgroup with persistent GI symptoms even on a strict gluten-free diet. This situation frequently frustrates patients and their families, since they could be keeping a gluten-free diet and the continuous questioning about diet adherence can develop into a stressful relationship between children, families and medical providers [93].

**What we know**: Rome IV criteria recommends an accurate evaluation for CD in children with symptoms suggestive of diarrhea-predominant IBS. In some adults with CD, even on a gluten-free diet, GI symptoms could persist.

**What we do not know**: It is unknown if IBS in pediatric patients increases the risk for developing CD. Functional abdominal pain disorders and IBS are unlikely to be caused by CD.

## 6. Evidence to Recommend Gluten Elimination Diet as a Treatment in Patients with FAPD

Currently, a strict, life-long gluten-free diet implementation is the only treatment option for patients with CD [88]. For IBS patients with NCGS, there are no specific treatment guidelines currently available [1]. The presence of gluten in foods is common, and, even in gluten-free foods, minute amounts of gluten can be present. The threshold for gluten tolerance in patients with NCGS is unknown, and it seems that value is variable among subjects [82,86,87].

Patients with abdominal pain and bloating reported control of symptoms, and patients with diarrhea-predominant IBS were found to have an improvement in symptoms when following a gluten-free diet for 6 months [115]. When successful, gluten avoidance can significantly improve symptoms in a short a time as within a week from initiation of the gluten-free diet. Adult non-celiac individuals with IBS keeping a gluten-free diet reported worsening symptoms when blindly exposed to gluten (68%) in comparison to patients that blindly received gluten-free placebo (40%) [52]. Other clinical trials in adults have supported the concept that gluten challenge causes symptoms in IBS [116,117,118]; wheat challenge causes symptoms in IBS [51]; and IBS symptoms with HLA DQ2-8 genetic haplotype responds to the gluten-free diet [73,115]. In a second trial by Biesikierski et al. patients with IBS with symptom resolution after a gluten-free diet were challenged with low-dose gluten (2 g per day), high-dose gluten (16 g per day) or whey protein (16 g per day) after keeping a low FODMAP diet for 2 weeks. The gluten challenge did not cause GI symptoms, but all challenged evoked neurological symptoms. There was no difference between different doses of gluten [119].

In patients self-diagnosed as NCGS/NCWS, before they start a gluten-free diet, it is recommended that they are tested to rule out CD [82]. Gluten-free diets with lower caloric and fiber concentrations can cause nutritional deficiencies and result in increased total and saturated fat intake. They can also contain lower amounts of folate, niacin, cobalamin, vitamin A, vitamin E, phosphorus, calcium, zinc, and selenium compared to gluten-rich foods [120,121,122,123]. The small intestine and fecal microbiota composition are affected, as seen with a reduction of beneficial bacteria like *Firmicutes*, when gluten is restricted in the diet [120,121,122,123]. Changes in intestinal microbiota and physiology after following a gluten-free diet could potentially enhance reactivity and sensitivity to gluten and/or wheat re-introductions [1]. To prevent inadequate nutrition, a gluten-free diet has to be medically indicated; it is also vital to have proper evaluation, guidance, and supervision by an experienced dietitian. Other food sensitivities and/or intolerances should be evaluated if GI symptoms persist after at least 6 weeks of a supervised gluten-free diet in patients with suspected NCGS/NCWS [55]. It is probable that other foods rich in FODMAPs could be causing symptoms, and an exclusion trial may be beneficial [1].

## 7. Conclusions

Consumption of certain foods, particularly gluten, is frequently associated with the development and persistence of FGIDs and FAPDs in adults and children. It is difficult to distinguish clear differences between gluten exclusion and improvement of symptoms related solely to functional disorders. Even more complicated is the potential role of immunity and chronic inflammation in FGIDs. CD and NCGS/NCWS are common and their incidence is increasing. These disorders occur in conjunction with FAPDs to some extent and are potentially associated. Currently, in pediatric patients, a gluten-free diet needs to be medically indicated and closely monitored by a healthcare provider, since it can lead to other nutritional deficiencies. Further research is imperative to better delineate the prevalence and mechanisms of gluten/wheat sensitivity and their significance before recommending gluten restriction in children with FAPD. 

## Figures and Tables

**Table 1:** Rome IV classification for functional gastrointestinal disorders for children and adolescents [10].

○ Functional nausea and vomiting disorders ▪ Cyclic vomiting syndrome ▪ Functional nausea and functional vomiting ▪ Rumination syndrome ▪ Aerophagia ○ Functional abdominal pain disorders ▪ Functional dyspepsia Postprandial distress syndrome Epigastric pain syndrome ▪ Irritable bowel syndrome (IBS) IBS with predominant constipation (IBS-C) IBS with predominant diarrhea (IBS-D) IBS with mixed bowel habits (IBS-M) IBS unclassified (IBS-U) ▪ Abdominal migraine ○ Functional abdominal pain—not otherwise specified ▪ Functional defecation disorders ▪ Functional constipation ▪ Non-retentive fecal incontinence

**Table 2:** Prevalence of functional gastrointestinal disorders (FGIDs) and functional abdominal pain disorders in children from United States and Colombia using the Rome IV classification [19,20].

Type of FGID	United States *n* (%)	Colombia *n* (%)
Any FGID	240 (25.00%)	755 (21.20%)
Functional Dyspepsia—Postprandial Distress Syndrome	69 (7.20%)	97 (2.70%)
Functional Dyspepsia—Epigastric Pain Syndrome	4 (0.40%)	11 (0.30%)
Irritable Bowel Syndrome	49 (5.10%)	83 (2.30%)
Abdominal Migraine	11 (1.10%)	18 (0.50%)
Functional Abdominal Pain—Not Otherwise Specified	30 (3.10%)	85 (2.40%)
